# Marginal public health gain of screening for colorectal cancer: modelling study, based on WHO and national databases in the Nordic countries

**DOI:** 10.1111/j.1365-2753.2012.01845.x

**Published:** 2013-04

**Authors:** Johann A Sigurdsson, Linn Getz, Göran Sjönell, Paula Vainiomäki, John Brodersen

**Affiliations:** 1 Professor, Department of Family Medicine, University of Iceland and Centre of Development, Primary Health Care of the Capital AreaReykjavik, Iceland; 2 Professor, General Practice Research Unit, Department of Public Health and General Practice, Norwegian University of Science and Technology (NTNU)Trondheim, Norway and Landspitali University Hospital, Reykjavík, Iceland; 3 General practitioner, Kvartersakuten Mörby CentrumDanderyd, Sweden; 4 Clinical Teacher, Family Medicine, University of Turku, Turku University HospitalTurku, Finland; 5 Associate Research professor, Research Unit and Section for General Practice, Department of Public Health, University of CopenhagenCopenhagen, Denmark

**Keywords:** colorectal cancer, mass screening, haemoccult, premature death, prioritization, public health

## Abstract

**Aims:**

To estimate the potential gain of national screening programmes for colorectal cancer (CRC) by stool occult blood testing in the Nordic countries, with comparative reference to the burden of other causes of premature death.

**Methods:**

Implementation of national screening programmes for CRC was modelled among people 55–74 years in accordance with the 2011 Cochrane review of biannual screening, using the faecal occult blood test (FOBT) for 10 years, resulting in 15% relative risk reduction in CRC deaths among all those invited [intention-to-treat; relative risk 0.85; confidence interval (CI) 0.78 to 0.92]. Our calculations are based on the World Health Organization and national databanks on death causes (ICD-10) and the mid-year number of inhabitants in the target group. For Finland, Denmark, Norway and Sweden, we used data for 2009. For Iceland, due to the population's small size, we calculated mean mortality for the period 2005–2009.

**Results:**

Invitation to a CRC screening programme for 10 years could influence 0.5–0.9% (95%CI 0.4–1.2) of all deaths in the age group 65–74 years. Among the remaining 99% of premature deaths, around 50% were caused by lung cancer, other lung diseases, cardiovascular diseases and accidents, with some national variations.

**Conclusions and implications:**

Establishment of a screening programme for CRC for people aged 55–74 can be expected to affect only a minor proportion of all premature deaths in the Nordic setting. From a public health perspective, prioritizing preventive strategies targeting more prevalent causes of premature death may be a superior approach.

## Introduction

The overall benefit of cancer screening programmes is generally debated [1–4], and colorectal cancer (CRC) is no exception [4–6]. Nevertheless, CRC screening is currently being introduced in many countries [7], assuming that the programme will entail important health benefits. It is essential that authorities and politicians are well-informed regarding the gains that can be expected from such a programme. On the basis of a recently updated Cochrane meta-analysis [8], the potential gain from CRC screening can be modelled. Here, we present and reflect upon such an analysis, with reference to data from the five Nordic countries.

CRC is a leading cause of cancer mortality in the Western world [8]. The precancerous stage is usually manifested in small intestinal polyps, and it can take years or decades for polyps to develop into life-threatening cancer. The clinical rationale behind CRC screening is that early detection (by screening) and extirpation of clinically asymptomatic polyps leading to occult bleeding will reduce the mortality of CRC. The obtained reduction in disease-specific mortality has traditionally been interpreted as equivalent to ‘saving lives’.

Different technologies can be used when screening for CRC: faecal occult blood test (FOBT), flexible sigmoidoscopy, colonoscopy, CT, MRI and a range of molecular markers. The best available evidence exists in relation to FOBT screening, and this modality has been the screening test offered to the citizens when national programmes have been implemented. In the present paper, we have therefore modelled the potential impact of FOBT-screening.

Starting in the 1980s, four influential randomized controlled trials (RCTs) of CRC screening were conducted in the regions of Fyn (Funen), Denmark; Gothenburg, Sweden; Minnesota, USA and Nottingham, UK [8]. The conclusion from each of these studies was that the disease-specific mortality from CRC could be reduced by systematic screening for blood in the stools with FOBT (Haemoccult, or Haemoccult II), followed by endoscopy, usually colonoscopy. The participants' ages typically ranged from 45 to 75 years. In the European regions, the participation rate was around or below 67%.

The 2011 Cochrane report [8] presents a comprehensive meta-analysis of existing knowledge regarding the effects of CRC screening. It is mainly based on the four above-mentioned RCTs and further follow-up of these. The analysis includes a total of 320 000 people, with follow-up for 8 to 18 years. The review concludes that invitation to participate in biannual screening for 10 years reduced the relative risk of dying from CRC by 15% in the target group.

**Figure 1:**
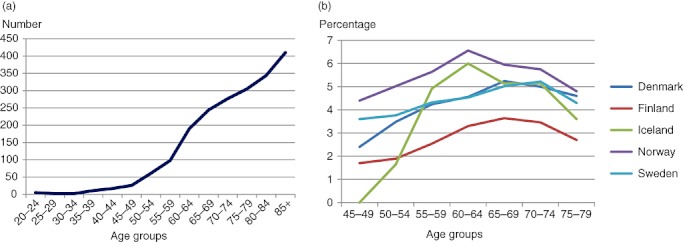
(a) Total number of deaths by colorectal cancer in Denmark 2009, by age group (Statistics Denmark 2011; statistikbanken.dk). (b) Mortality of colorectal cancer in the Nordic countries as a proportion of all deaths in each age group (WHO, European Detailed Mortality Database, http://data.euro.who.int/dmdb/, Denmark, year 2006, Sweden, 2008, and the others, 2009).

Death by CRC is rare before the age of 50, but increases rapidly thereafter (Fig. 1a). When arguing for cancer screening, opinion leaders typically provide politicians and the public with national figures showing the total number of deaths from the particular cancer in question, regardless of age. In the case of CRC, these figures have been accompanied by a claim that screening will reduce CRC deaths by 20–25% [9,10]. The resulting estimate of lives that can potentially be saved by CRC screening looks compelling. However, these calculations overestimate the benefit because they disregard the fact that most CRC deaths occur in older age groups not invited to screening, and empirical evidence is lacking on whether a person remains protected from CRC death long after exiting the screening programme [5]. The numerical gains from CRC screening programmes can therefore not be extrapolated to the total number of CRC deaths in a nation.

Until 2008, the Cochrane reviews did not assess the impact of CRC screening with respect to *all-cause mortality*. But since then, updated reviews have documented that the all-cause mortality did not decrease in the age groups screened [8,11].

The age-standardized death rates of CRC have decreased in the Nordic countries during the last decades [World Health Organization (WHO) and national databases, Box 1], even in the absence of national screening programmes. A similar phenomenon has been reported for breast cancer [12]. However, in both Norway and Denmark, national CRC screening programmes are currently being planned/implemented [13,14]. In Finland, screening for CRC started in 2004 among people aged 60–64 in 22 of 444 municipalities. By 2007, the programme encompassed 175 municipalities for the age group 60–67 years [15]. In Sweden, people aged 60–69 in Stockholm have been invited for CRC screening since 2008 [16]. CRC screening has also been proposed in Iceland for the age group 55–70 years [9].

**Box 1 International and Nordic databases on populations and causes of death**

- World Health Organization Regional Office for Europe
  - European Detailed Mortality Database; http://data.euro.who.int/dmdb/
  - European Health for All Database (HFA-DB) http://data.euro.who.int/hfadb/
- Statistics Denmark http://statistikbanken.dk/
- SOTKAnet, Statistics and Indicator Bank 2005–2011, Finland; http://uusi.sotkanet.fi/portal/page/portal/etusivu/hakusivu?group=219
- Statistics Iceland; http://www.hagstofan.is
- Statistics Norway: http://www.ssb.no/helsetilstand_en/
- Statistics Sweden (population); http://scb.se
- National Board of Health and Welfare, Sweden (mortality); http://www.socialstyrelsen.se/statistik/statistikdatabas

The purpose of this study was to model the potential gain from screening for CRC in the Nordic setting, in terms of reduction in premature deaths. The analysis is based on the best available evidence regarding the effects of screening. In addition, we depict and reflect upon the contribution of CRC mortality to the total panorama of premature death among people in the age group 65 to 74 years.

## Study populations and methods

Our model is based on the 2011 Cochrane meta-analysis of effect sizes and the analysis's recommendations regarding application of these findings for research and policy planning purposes [8,11]. The potential gain on a community level is based on an ‘intention-to-treat’ analysis. In accordance with the participation rate of around 70% documented in the Cochrane meta-analysis, we thereby model a 15% relative risk reduction in CRC mortality per year among asymptomatic individuals who are invited to bi-annual screening for occult stool blood with Haemoccult for a 10-year period [relative risk 0.85, confidence interval (CI): 0.78–0.92]. It was assumed that CRC deaths prevented by the screening programme would be distributed evenly over these 10 years. In accordance with the Cochrane review, our model presupposes that CRC screening will not affect all-cause mortality (fixed effects model: relative risk 1.0, CI: 0.99–1.02). The proportion of CRC deaths in relation to total deaths was calculated for different age sub-groups.

**Figure 2:**
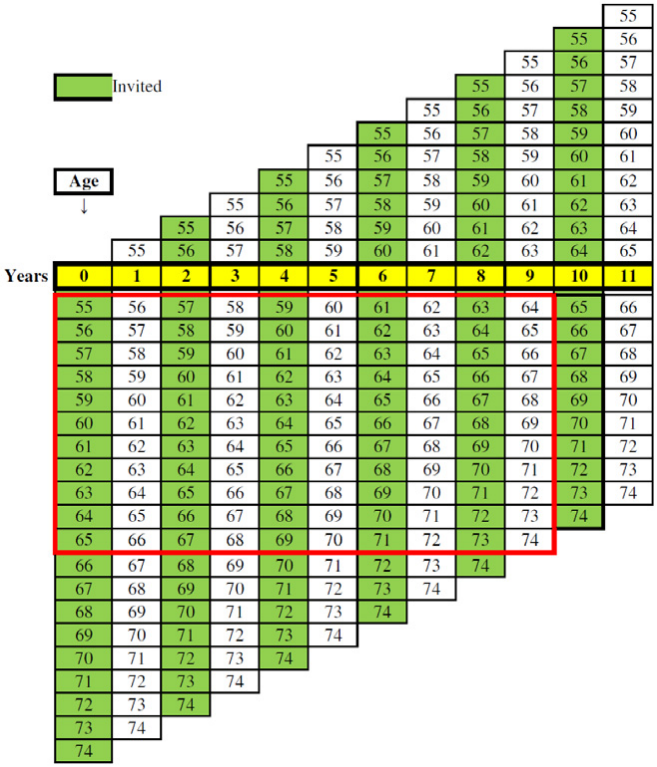
Illustration of a national screening programme, starting at a certain year zero, for colorectal cancer, with an invitation to all people aged 55–74. During the coming years, new groups of people will be included/invited when they turn 55 (above the yellow years bar), and others will exit when they pass age 74 (below the yellow years bar). The red box indicates the age groups that have been in the programme (invited and/or participating) for 10 years.

To be concrete, we envisage a screening programme starting at ‘year zero’ with an invitation for biannual screening for all individuals aged 55 to 74 years (Fig. 2). During the following years, those turning 75 will exit the invitation programme, and others turning 55 will be included. Thus, those who were 74 years at the start will be invited only once, those who are 72 at start twice, and so on. After 10 years, only those who were 55–65 at ‘year zero’ have been invited for 10 years. These people are then aged 65–74. Consequently, we examined the potential effects on mortality in this age group.

For the modelling analysis, we used data from the national databanks on statistics in the Nordic countries (Denmark, Finland, Iceland, Norway and Sweden, and the WHO mortality database, see Box 1). Data for mid-year population size and deaths occurring between the ages of 65 and 74 were extracted for 2009, except for Iceland, where, due to the population's small size, we calculated average prevalence figures for the period 2005–2009. The causes of death were classified on the basis of the short European list (Denmark, Iceland and Sweden) and the full version (Finland and Norway), according to the 10th edition of the International Health Organization's classification system (ICD-10). The number of deaths per year was used to calculate the potential gain of CRC screening according to the Cochrane review. We also assumed that the ratio between the various death causes and the total population (numbers pertaining to 2009, except for Iceland) would remain unchanged in the modelling period. For the purpose of sensitivity analyses, we used the highest (0.78 = 22%) and lowest (0.92 = 8%) relative risk reduction (RRR) in the confidence interval from the Cochrane review to estimate the highest and lowest potential benefits of screening. Premature death was defined as death before the age of 75. Premature death rate (PDR) was calculated as the number of deaths/1000 of inhabitants for the age group 65–74 years.

As screening for CRC had already started to some extent in Finland in 2004, a comparison was made of the PDRs, total deaths and deaths from CRC during the years 2004 and 2009.

The chi-square test was used for statistical comparison of category variables. Significance was defined as *P* < 0.05.

## Results

The total number of deaths attributed to CRC at any age in the Nordic countries is shown in Table 1. It appears that the standardized death rate of CRC is highest in Denmark and lowest in Finland.

**Table 1:** Midyear total population, total number of deaths and deaths by colorectal cancer in the Nordic countries in 2009, and total and age-standardized death rates (SDR) in 2009, except for Denmark, with SDR statistics from 2006

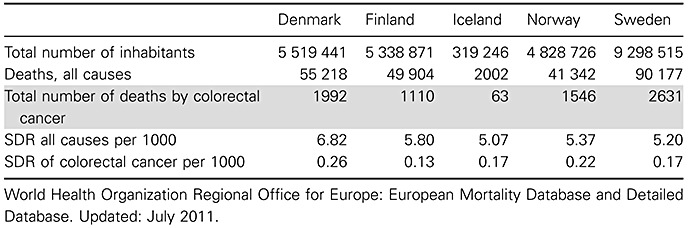

Death by CRC increases with age, as can be seen in Fig. 1a (only shown for Denmark). The same is also true for most other causes of death. The proportion of CRC deaths in relation to the total number of deaths increases after the age of 50, but declines again after age 75, as shown in Fig. 1b.

Table 2 shows the target (total mid-year) population after invitation to screening for 10 years, as well as absolute and relative figures for possible gain from screening in each country. It is evident that the screening programme can only affect a small proportion of all premature deaths (varying between 0.5% and 0.9%).

**Table 2:** Total (=target) populations,* all-cause mortality per year, mortality due to colorectal cancer (CRC), estimated absolute and relative reduction (%, and 95% CI within brackets) of deaths from CRC and deaths from other causes after invitation to CRC screening for 10 years among people 65–74 years old in the Nordic countries

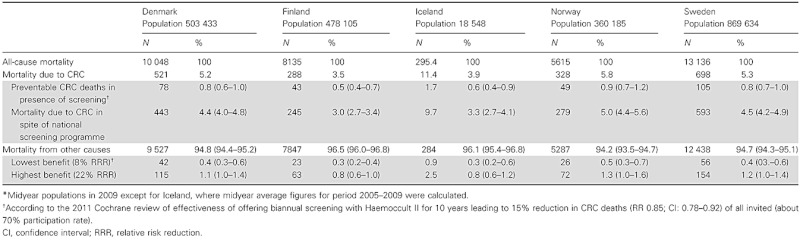

The PDR in the age group 65–74 years was highest in Denmark (19.96/1000 inhabitants), compared with 17.02 in Finland, 15.90 in Iceland, 15.59 in Norway and 15.11 in Sweden. The PDR in Finland in 2004 was somewhat higher than in 2009, that is 18.73/1000 inhabitants. CRC contributed 3.0% of all deaths in 2004 (95%CI: 2.6–3.4), which is a lower figure than in 2009 (see Table 2).

Tables 1 and 2 show that an invitation to screening for 10 years might possibly save 3.9% (78/1992) of the total number of CRC deaths per year in the age group 65–74 years in Denmark, 3.9% (43/1110) in Finland, 2.7% (1.7/63) in Iceland, 3.2% (49/1546) in Norway and 4.0% (105/2631) in Sweden.

Table 3 shows the most common causes of death in the age group 65–74 years. Malignant tumours dominate this picture. The high mortality rate due to lung cancer is particularly noteworthy. Death rates from cardiovascular diseases, accidents and suicide are higher in Finland than in Denmark, Norway and Sweden (*P* < 0.001).

**Table 3:** Main causes of premature deaths in the age group 65–74 years in the Nordic countries (diagnoses according to ICD-10 within brackets)*

	Denmark		Finland		Iceland		Norway		Sweden	
					
	Number		Number		Number		Number		Number	
Cause of death	*N* = 10048	%	*N* = 8135	%	*N* = 295.4	%	*N* = 5615	%	*N* = 13136	%
Malignant neoplasms total (C00-D48) in	4210	41.9	2845	35.0	134.4	45.5	2427	42.9	5606	42.7
Lungs, bronchi, larynx (C32-C34)	1192	11.9	640	7.9	42.6	14.4	617	11.0	1161	8.8
Colon-rectum (C18-C21)	521	5.2	288	3.5	11.4	3.9	328	5.8	698	5.3
Prostate (C61)	305	3.0	191	2.3	9.2	3.1	168	3.0	458	3.5
Breasts (C50)	303	3.0	174	2.1	9.8	3.3	127	2.3	302	2.3
Cardiovascular diseases (I00-I99)	2081	20.7	2975	36.6	87	29.5	1436	25.6	4005	30.5
Respiratory system diseases (J00-J99)	814	8.1	418	5.1	21.4	7.2	538	9.6	741	5.6
Gastrointestinal tract diseases (K00-K93)	522	5.2	427	5.2	8.4	2.8	172	3.1	476	3.6
Mental disorders incl. addiction (F00-F99)	287	2.9	132	1.6	3.2	1.1	106	1.9	240	1.8
Accidents, suicides and other external causes (V01-Y98)	137	1.4	494	6.1	9.2	3.1	192	3.4	508	3.9
Suicides (X60-X84)	85	0.3	94	1.2	1.6	0.5	47	0.8	141	11.1
Other causes of death	1997	19.9	844	10.4	31.8	10.8	744	13.3	1560	11.9

* The data refer to 2009 except for Iceland, where the average figures from 2005 to 2009 are calculated.

**Figure 3:**
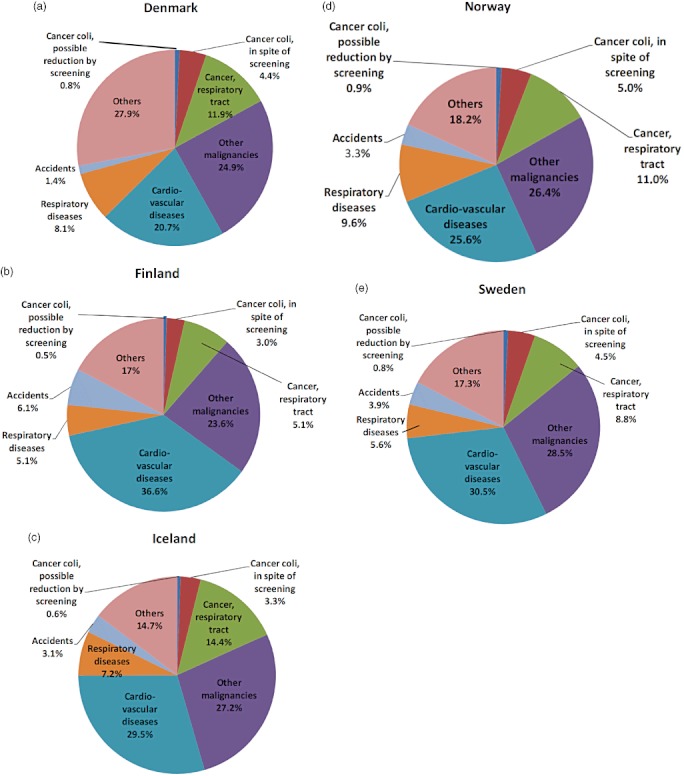
(a–e) Illustration of the potential for reducing mortality due to colorectal cancer after invitation to screening for 10 years, seen in comparison to premature deaths from other causes among people in the Nordic countries in the age group 65–74.

Figure 3a–e shows the proportions of various causes of premature death for the age range in question. As can be seen, the mortality from CRC is a small fraction of the total, and invitation to screening for CRC will not affect the cause of death for 99% of the individuals who die at this early age. In comparison, cancer of the lungs, other pulmonary diseases, cardiovascular diseases and accidents account for 42–54% of the deaths.

## Discussion

Invitation to CRC screening by way at occult stool blood testing for 10 years could reduce CRC deaths in people aged 65–74 by less than 1.0% of all deaths. This finding should be considered in light of the fact that around 50% of all premature deaths around this age are caused by diseases where increased efforts can still be directed towards known and potentially modifiable risk factors.

### Type of screening tools

Most countries with CRC screening still rely on screening for occult blood in stools [7]. Despite substantial technological progress in recent decades, it has so far not been documented decisively that other methods, such as colonoscopies for everyone, DNA testing of stool samples or computed tomography of the colon, provide better results. All of these methods have their advantages and limitations [17–19]. Four randomized controlled trials on CRC screening using flexible sigmoidoscopy have recently been conducted [19–22]. Two of these have so far only reported findings from the prevalence round [20,21]. A third study showed a relative risk reduction in CRC mortality of 23% in the intention-to-treat analysis [19]. The fourth study revealed a non-significant CRC mortality reduction in the intention-to-treat analysis [22]. If we had based our modelling study on the 23% mortality reduction reported by Atkin *et al*. [19], the conclusion would not have differed substantially, as shown by our sensitivity analyses. Furthermore, a recent study [6] indicates that the relative risk reduction in CRC mortality found in the updated Cochrane review's meta-analysis [8] is overestimated because of biases in the RCT studies upon which the Cochrane review is based.

We were not able to estimate prevented CRC deaths attributable to screening studies by other modalities than FOBT and flexible sigmoidoscopy, because no data on CRC mortality reduction have been reported in these settings.

There are small differences among the Nordic countries regarding the relative death rates by CRC, but the situation currently seems best in Finland. In that setting, it may therefore be important to prioritize further interventions directed towards cardiovascular diseases and violent deaths/accidents.

To extend our perspective to a wider Western European context, it can be noted that the reported proportion of CRC deaths in several European countries are closely comparable with the Nordic figures (expressed as the percentage of all deaths in the age groups 65–74 years); in France, it is 4.5%, Germany 4.6%, Ireland 4.9%, the Netherlands 5.5% and in the United Kingdom 4.1% (WHO databank, Box 1). Our analysis thereby has considerable relevance beyond the Nordic region.

### Strengths and weaknesses of the study

The validity of this study evidently depends on the correctness of coding of death causes in the national registries [23]. We see no reason to believe there are systematic errors in the coding of CRC deaths, compared with other conditions.

Modelling studies are an important step in the preparation of concrete healthcare action plans [24]. Their inherent weakness is evidently the application of retrospective data for the purpose of predicting, which is a problem associated with all evidence-based preventive planning. Since the age-standardized death rate by CRC has decreased during the last decades in the countries studied, it is possible that our model will attribute more gain to the screening procedure than is strictly warranted, since some decrease in death from CRC may have other explanations, including improved therapy for cancer detected at an early symptomatic stage.

As stated in the Cochrane meta-analysis, the maximal effect of a systematic screening programme for CRC will not be achieved until everyone has been invited to participate for 10 years. Our model is therefore based on such evidence. It is however likely that some gain can be achieved even by those invited for shorter periods, that is 1–9 years (as illustrated in Fig. 2). This might lead to underestimation of the total benefit. However, the possible benefit in the age group 55–64 cannot be greater than that estimated in the age group 65–74 in this study.

It can be assumed that a CRC screening programme will increase the number of colonoscopies and polypectomies. This might, despite the current lack of evidence of increased survival, improve life expectancy several years beyond the screening programme [8]. Therefore, the results could be underestimated if we only count the benefit of screening until the exit year of the invitation for 10 years. However, although the number of colonoscopies substantially increases with follow-up of positive stool samples, the predictive value of polypectomies with respect to CRC is low: 0.7% according to figures calculated from a study by Citarda *et al*. [25,26]. This means that at least one polypectomy must be performed per 145 individuals in order to prevent one case of CRC.

### Participation rates

To obtain good participation rates in public screening programmes, health authorities are likely to encourage people to believe in the value of screening. Consequently, the information to the public may be biased towards the potential gains. Clinical guidelines advocate a neutral stance; they argue that participation in screening programmes should be an ‘informed uptake’ after information about both benefits and harms [1,27–29]. Information explicitly mentioning harms might decrease participation rates [29], but this has so far not been formally documented [30]. The impact of balanced information might also be influenced by the nature of the screening test in question. It should be noted that if participation falls below 60%, it is uncertain whether one can expect any public health gain from CRC screening at all.

### Prioritization in preventive health care

Considering the small contribution of CRC deaths to the total number of premature deaths (Fig. 3), one may wonder why screening for CRC has attracted so much attention and resources. A partial explanation may be that many of the competing causes of premature death, including smoking, alcohol and drug-related diseases, have a clear social gradient. This means that they occur relatively less frequently in affluent segments of society [31]. In comparison, CRC lacks a clear social gradient, and the disease may more easily attract the personal attention of politicians and health policy planners.

## Conclusion

Based on the most recent Cochrane evidence, our modelling represents an argument for critically reflecting on the value of CRC screening as part of the preventive medical portfolio in the Nordic countries. Systematic screening for CRC can favourably impact only a small fraction of all causes of premature death. From a public health perspective, it may be better to prioritise other preventive measures, targeting commoner causes of premature death.
